# Multiple-Brain Connectivity During Third Party Punishment: an EEG Hyperscanning Study

**DOI:** 10.1038/s41598-018-24416-w

**Published:** 2018-05-01

**Authors:** A. Ciaramidaro, J. Toppi, C. Casper, C. M. Freitag, M. Siniatchkin, L. Astolfi

**Affiliations:** 1 Department of Computer, Control, and Management Engineering, Univ. of Rome “Sapienza”, Rome, Italy, 00185 Italy; 2 Department of Child and Adolescent Psychiatry, Psychosomatics, and Psychotherapy, Goethe-University, Frankfurt/M, 60528 Germany; 3 Neuroelectrical Imaging and Brain Computer Interface Laboratory, Fondazione Santa Lucia IRCCS, Rome, Italy, 00179 Italy; 4 Institute of Medical Psychology and Medical Sociology, University of Kiel, Kiel, 24113 Germany

## Abstract

Compassion is a particular form of empathic reaction to harm that befalls others and is accompanied by a desire to alleviate their suffering. This altruistic behavior is often manifested through altruistic punishment, wherein individuals penalize a deprecated human’s actions, even if they are directed toward strangers. By adopting a dual approach, we provide empirical evidence that compassion is a multifaceted prosocial behavior and can predict altruistic punishment. In particular, in this multiple-brain connectivity study in an EEG hyperscanning setting, compassion was examined during real-time social interactions in a third-party punishment (TPP) experiment. We observed that specific connectivity patterns were linked to behavioral and psychological intra- and interpersonal factors. Thus, our results suggest that an ecological approach based on simultaneous dual-scanning and multiple-brain connectivity is suitable for analyzing complex social phenomena.

## Introduction

**Figure 1:**
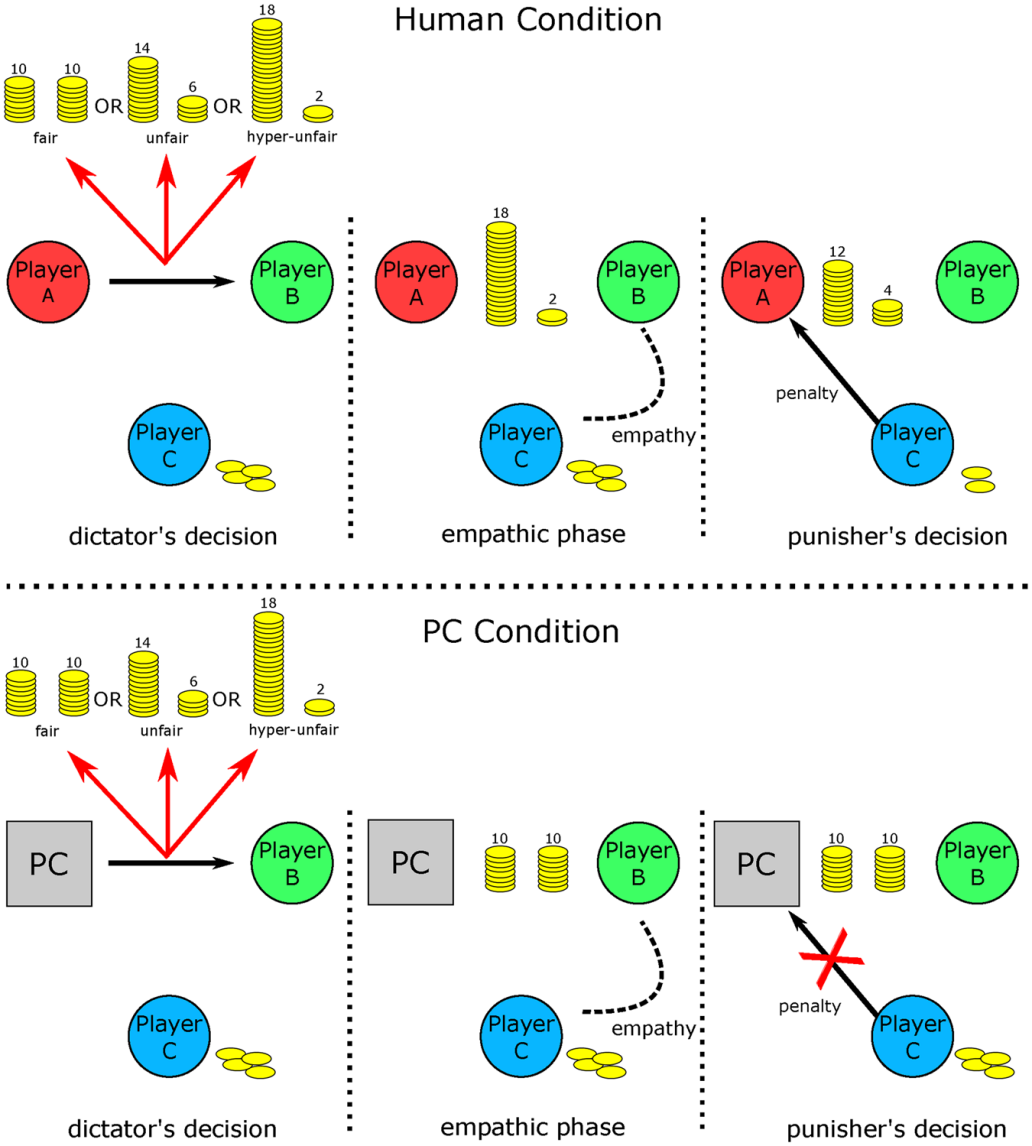
Schematic of TPP game. TPP involves three subjects: player A (the dictator), player B (the receiver), and player C (the observer, who in turn can act as a punisher). Player A has an initial endowment of 20 points, which he can share with player B, selecting among three conditions: fair (10 for A and 10 for B), unfair (14 for A and 6 for B), and hyperunfair (18 for A and 2 for B). Player A’s decision is shown simultaneously to all players (empathic phase). Player C observes the dictator’s decision and can use part, or all, of his endowment (4 points) to punish Player A’s behavior. For every punishment point invested by Player C, 3 points are subtracted from Player A’s payoff, and 1 point is added to Player B’s payoff.

A social group is based on the ability of individuals to share the affective states of others through empathy, which allows others-oriented prosocial behavior. Compassion is defined as an empathic reaction that induces feelings concern for another person’s suffering, in association with an approach and motivation to help^1^. A particular form of prosocial behavior that is related to compassion is *altruistic punishment*, in which individuals tend to (costly) punish unfair behavior and violators of social norms, even when norm violation occurs against strangers^2,3^. In this study, compassion was examined in a true social interaction using the third party punishment (TPP) paradigm, which permits ecological real-time measurements of both brains that are involved in an empathic experience and the consequent prosocial behavior. TPP is a particular form of the dictator game, in which a dictator splits a sum of money with a receiver. A third party (the punisher) observes the interaction between the dictator and receiver and can decide to punish the former’s behavior. Thus, this game elicits and—indirectly, through punishing behavior—quantifies the empathy that arises between the punisher and the receiver and that leads the punisher reacting to the (fair or unfair) economic treatment that is imposed by the dictator onto the receiver (see Fig. 1). In fact, the human innate tendency to prefer equal shares (or the aversion to inequity) elicits an altruistic sharing behavior^4^. Different theories emphasize a close link between empathy and altruism. In particular, the empathy–altruism hypothesis^5^ has posited that empathy promotes altruistic motivation toward a person in need, namely the motivation to help the person for whom empathy is felt. Without empathic feeling, altruism can hardly occur. Consequently, justice sensitivity is strictly related to the experience of unfairness, which, when concerning others, cannot exist without feeling compassion^4–9^.

Neuroimaging studies have examined compassion and altruistic punishment using monetary games or legal scenarios in various social contexts (acting alone or in a group or using a parochial dimension), implicating a large-scale network that comprises the salience network, the mentalizing system, and part of the central executive network during this complex form of social behavior^10^. However, these studies used a single-person paradigm, focusing on a single mind (mostly the punisher) and neglecting the interpersonal dynamics and reciprocal influence between the ‘interacting’ individuals who are typically involved in prosocial behavior. Recently, a new conceptual and methodological framework was recommended in analyzing the neural basis of human social interaction: *two-person neuroscience* (2PN), an approach for studying two interacting persons at the same time, that focuses on the dyad rather than the individual^11^. This approach exploits the simultaneous neuroimaging of two or more subjects (commonly referred to as *hyperscanning* or dual scanning; for a review, see^11,12^). In the framework of 2PN, Astolfi and colleagues proposed an approach that integrates neuroelectrical hyperscanning and advanced methods to estimate brain functional connectivity, allowing data that are simultaneously recorded from two interacting persons to be analyzed jointly in examined real social interactions. Hyperconnectivity, or multiple-brain connectivity, is defined as the study of temporal correlations between signals that are derived from the brain regions of various subjects during their interaction^12^. The basis of this technique is that the dyad (or group) of interacting subjects is a complex system, each component of which (i.e., each subject) influences and is influenced by the others, which is reflected by coordinated neural activity. The feasibility of multiple-brain connectivity in characterizing and predicting cooperative behavior, even before it is overtly expressed, was demonstrated by EEG-based studies, in which subjects simultaneously engaged in a card game^13^ or in tasks that were related to game theory^14–16^. These studies provided evidences that mathematical indices that are derived from multiple-brain modeling can discriminate cooperative and individualistic behaviors. To the best of our knowledge, no hyperscanning or multiple-brain connectivity study has focused on compassion or altruistic behavior before our preliminary study^17^ which proved the feasibility of this approach to capture the empathic interaction between a single pair of subjects.

**Figure 2:**
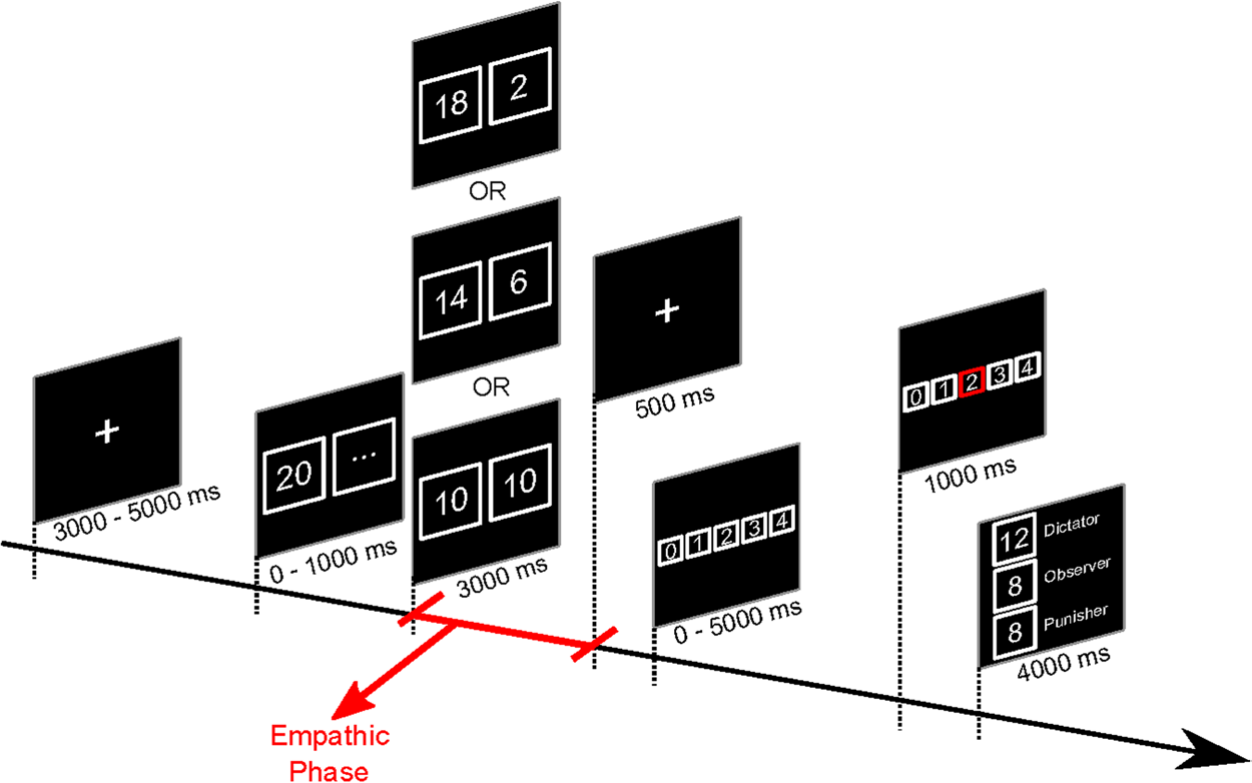
Timeline of the Third-Party Punishment paradigm for one block. This timeline is equal in the PC and human conditions. First, a fixation cross appears on the screen for a randomly selected period from 3000–5000 ms. Then, Player A is asked to make his decision among three alternatives (fair: 10 points to player A, 10 to player B; unfair: 14 points to player A, 6 to player B; hyperunfair: 18 points to player A, 2 to player B) in less than 1 second. Player A’s decision is then shown to all players for 3 seconds (empathic phase). Player C is then asked to select 0 to 4 punishment points within 5 s. For every punishment point invested by Player C, 3 points are subtracted from Player A’s payoff, and 1 point is added to Player B’s payoff. The trial ends, showing (4 seconds) a window containing the scores of the current trial. This scheme was repeated for 210 trials (half for human and half for PC condition), divided into blocks of 30 trials each.

Here, we report the results of an exploratory multiple-brain connectivity study in the EEG hyperscanning setting to determine the neuroelectrical correlates of compassion and altruistic behavior, focusing on the empathic interaction between players B and C (the receiver and observer/punisher) by means of a dual perspective in which the dyadic interaction between the two brains is measured simultaneously. A combination of multiple-brain connectivity and specific indices that are derived from graph theory was used to analyze the empathic reaction (compassion) that arose between the punisher and receiver during the observation of fair or unfair behavior in an economic exchange (Figs 1 and 2). Further, we examined whether compassion can predict altruistic punishment, as a prosocial behavior that is consequent to the empathic experience. Finally, because empathy is modulated by various factors^18^, we tested whether interindividual differences explain multifaceted prosocial behavior. To this end, we proposed the following hypotheses: i) Multiple-brain (and, in particular, interbrain) connectivity is modulated by the fairness of the dictator’s decision: i.e., compassion can be measured through a quantitative index that is positively related to the dictator’s behavior; ii) differences in multiple-brain connectivity arise in relation to the agency (human vs nonhuman condition): i.e., compassion can be measured through a qualitative index that is related to a human or nonhuman agent (PC); iii) there is a relationship between multiple-brain connectivity and the punisher’s behavior: i.e., quantitative indices predict punishment scores.

## Results

This multiple-brain connectivity study involved 42 healthy male volunteers, organized into 21 dyads that performed the TPP paradigm. EEG signals were recorded simultaneously from each dyad using 60 EEG channels for each subject. The two participants acted as receiver and punisher; the dictator was, instead, a confederate. For details on the experiment, see the Methods section below.

### Behavioral Data

**Figure 3:**
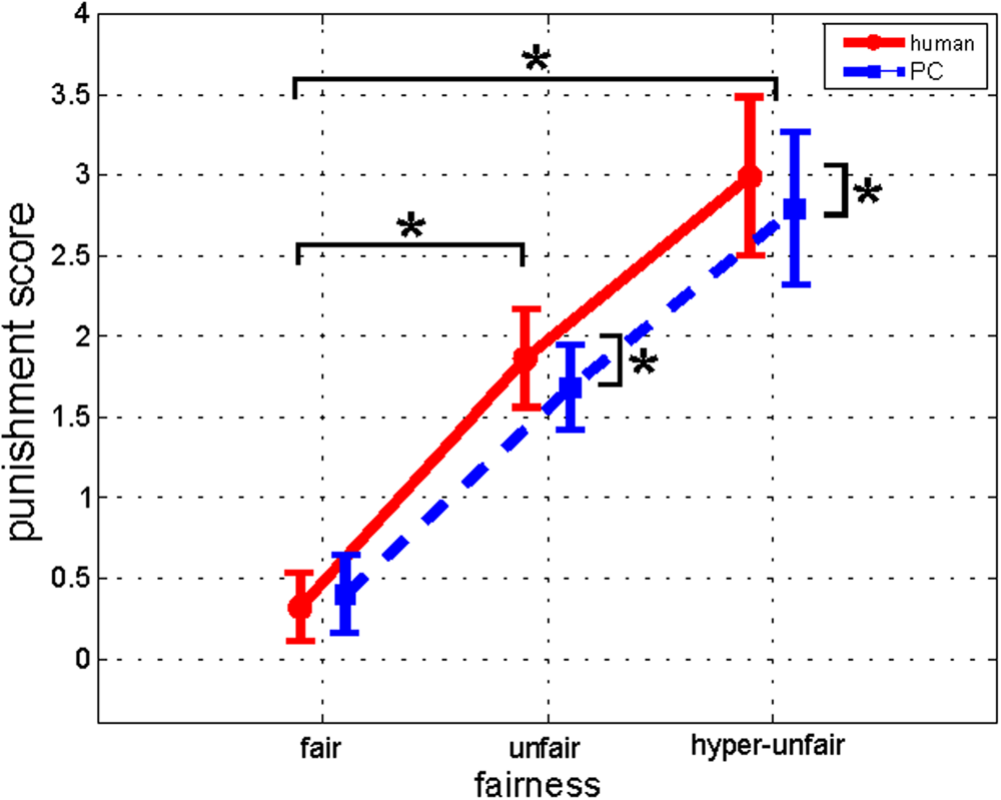
Analysis of behavioral data. Results of two-way repeated measures ANOVA considering punishment score as the dependent variable and AGENCY (human, PC) and FAIRNESS (fair, unfair, hyperunfair) as the main within-group factors (F = 4.37, p = 0.019). The bars represent the 95% confidence interval. *Indicates statistical differences between the corresponding levels (Neumann-Keuls post hoc test, p < 0.05).

Two-way repeated measures ANOVA was used to analyze the punishment scores to determine the *fairness* of the dictator’s decision (fair, unfair, hyperunfair) and the dictator’s *agency* (human or PC). The ANOVA results are reported in Fig. 3. In particular, we found a significant effect of the factor fairness [d.f. = (2.40), p < 0.00001, F = 92.44] and the interaction fairness x agency [d.f. = (2.40), p = 0.019, F = 4.37]. Specifically, the punishment score increased with greater unfairness of the dictator’s decision. Moreover, the punishment score was significantly higher in the human condition with respect to the PC for unfair and hyper-unfair conditions (Newman-Keuls post hoc test p < 0.05).

Next, we examined the correlation between the punishers’ behavior and their scores on self-reported questionnaires (empathy quotient [EQ], impression scale [IMP-S], and altruism scale [NEO-PI-R]), administered after the EEG session. In the correlation analyses between punishment score and questionnaire responses, there was a negative correlation between IMP-S and the human hyperunfair punishment. A good impression of the dictator by the punisher correlated negatively with the scores that were used to punish the dictator (r = −0.51 and p = 0.018, power = 0.53). No association between IMP-S and the PC hyperunfair punishment was observed (r = −0.33 and p = 0.14, power = 0.23). Also, NEO-PI-R (altruistic behavior) was positively linked to the human hyperunfair punishment (r = 0.45 and p = 0.04, power = 0.41), but NEO-PI-R and the PC hyperunfair punishment were not associated (r = 0.32 and p = 0.16, power = 0.22). There was no correlation between EQ (empathy) and the human hyperunfair punishment (r = −0.31 and p = 0.17, power = 0.21) or between EQ and the PC hyperunfair punishment (r = −0.12 and p = 0.6, power = 0.07).

### Multiple-brain connectivity and related indices

**Figure 4:**
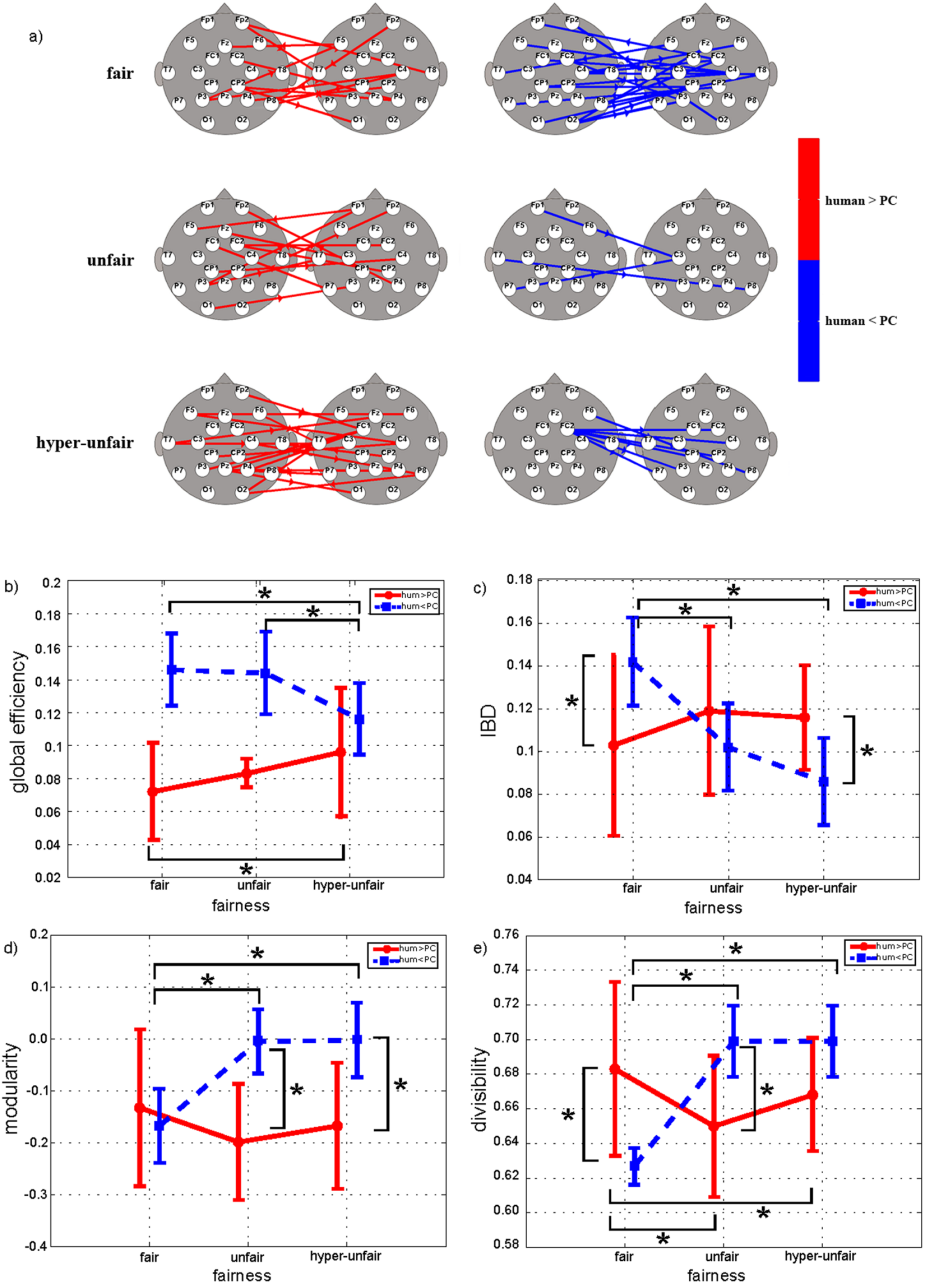
(**a**) Statistical interbrain connectivity patterns in theta band. Statistical interbrain connectivity patterns comparing human vs PC conditions in the theta band (group analysis, paired t-test; p < 0.05 FDR-corrected). The patterns were reconstructed for the three fairness levels separately. Red arrows indicate human > PC, and blue arrows indicate human < PC. b,c,d,e) Results of two-way ANOVA on global efficiency (p = 0.00024, F(2,28) = 11.39) (panel b), interbrain density (p = 0.0003, F(2,28) = 11.13) (IBD - panel c), modularity (p = 0.016, F(2,28) = 4.84) (panel d), and divisibility (p = 0.0007, F(2,28) = 9.62) (panel e) computed for connectivity networks in theta band. *Indicates statistical differences as confirmed by Neumann-Keuls post hoc test (p < 0.05).

**Figure 5:**
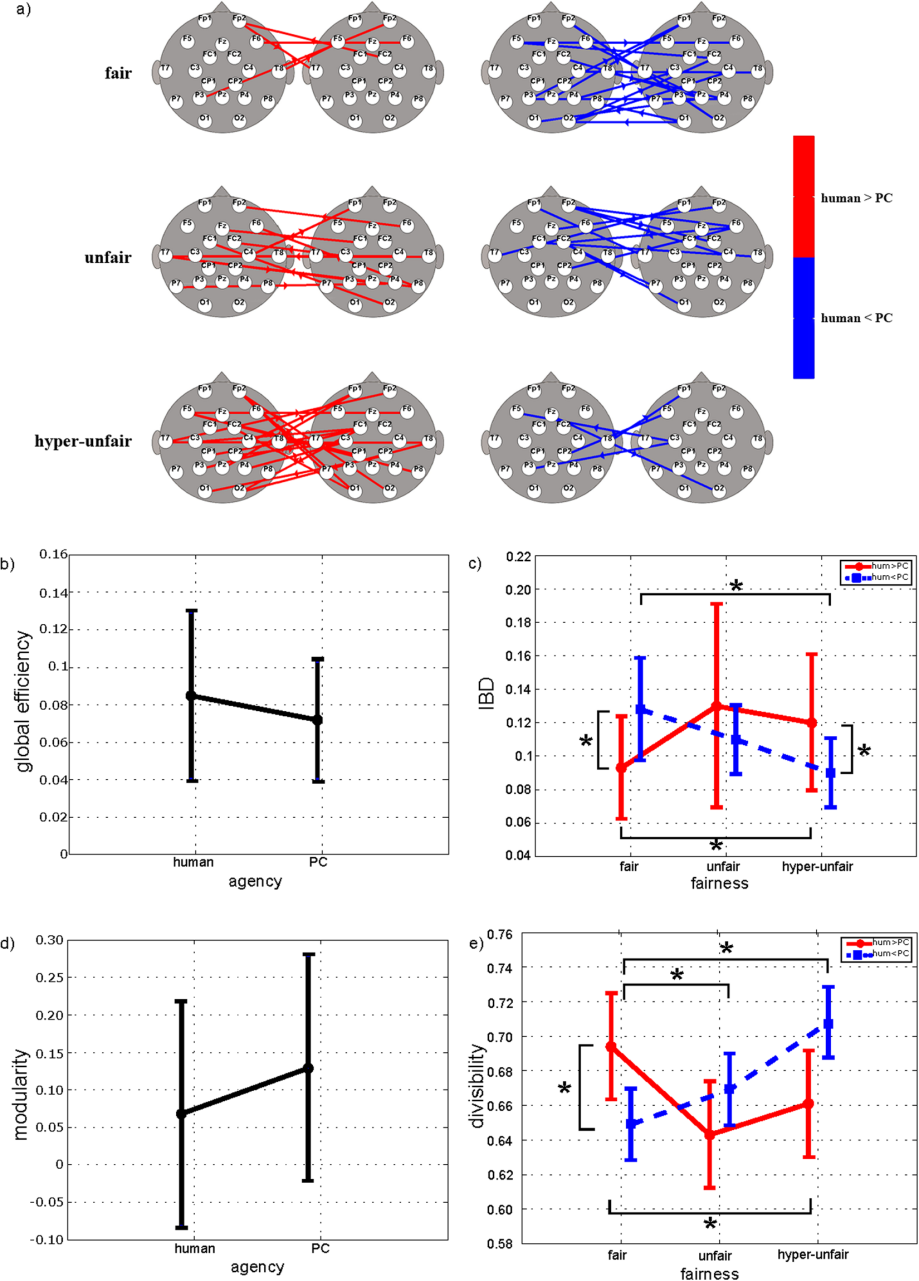
(**a**) Statistical interbrain connectivity patterns in alpha band. Statistical interbrain connectivity patterns comparing human vs PC conditions in the alpha band (group analysis, paired t-test; p < 0.05 FDR-corrected). The patterns were reconstructed for the three fairness levels separately. Red arrows are for human > PC, and blue arrows are for human < PC. b,c,d,e) Results of two-way ANOVA on global efficiency (p = 0.032, F(1,14) = 5.64) (panel b), interbrain density (p = 0.0095, F(2,28) = 5.52) (IBD - panel c), modularity (p = 0.006, F(1,14) = 10.47) (panel d), and divisibility (p = 0.0062, F(2,28) = 6.13) (panel e) computed for connectivity networks in alpha band. *Indicates statistical differences as confirmed by Neumann-Keuls post hoc test (p < 0.05).

**Figure 6:**
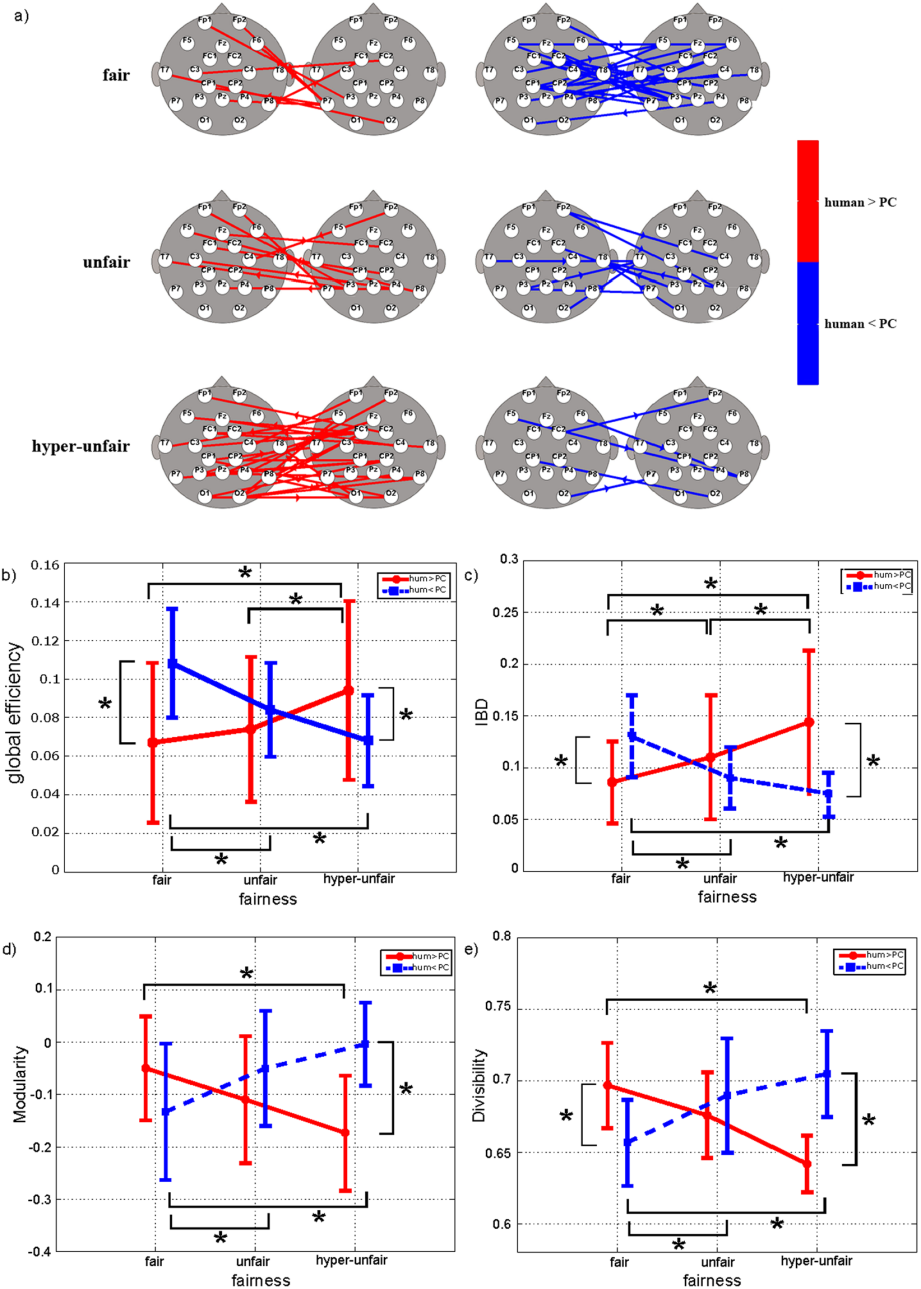
(**a**) Statistical interbrain connectivity patterns in beta band. Statistical interbrain connectivity patterns comparing human vs PC conditions in the beta band (group analysis, paired t-test; p < 0.05 FDR-corrected). The patterns were reconstructed for the three fairness levels separately. Red arrows are for human > PC, and blue arrows are for human < PC. b,c,d,e) Results of two-way ANOVA performed on global efficiency (p = 0.0001, F(2,28) = 19.04) (panel c), interbrain density (p = 0.00001, F(2,28) = 20.83) (IBD - panel b), modularity (p = 0.00021, F(2,28) = 11.66) (panel d), and divisibility (p = 0.00009, F(2,28) = 13.16) (panel e) computed for connectivity networks in beta band. *Indicates statistical differences as confirmed by Neumann-Keuls post hoc test (p < 0.05).

EEG signals that were simultaneously recorded from each dyad and segmented in the TPP empathic period (see Figs 1 and 2) were preprocessed and then subjected to connectivity analysis as a unique dataset. Multiple-brain connectivity patterns were estimated for each dyad in the six combinations of experimental conditions—3 fairness levels (fair, unfair, hyperunfair) X 2 agent levels (human, PC). Group connectivity patterns were extracted by comparing human and PC conditions separately for the three fairness levels and four frequency bands (theta, alpha, beta, and gamma). Figures 4a, 5a and 6a show the statistical patterns for the theta, alpha, and beta bands. Graph theory was used to characterize the properties of multiple-brain connectivity patterns, implementing the most frequently used global indices (*global efficiency, local efficiency, path length*, and *clustering coefficient*) to describe the tendency of a network to communicate through clusters and to characterize the efficiency of communication between nodes. High values of *global efficiency, local efficiency*, and *clustering* and low values of *path length* were associated with highly interconnected networks. Further, we included indices with a specific relevance to multiple-brain networks (*interbrain density* [IBD], *divisibility*, and *modularity*) as measures of the interaction between the brain activity of two subjects, because they are sensitive to variations in brain-to-brain networks^15^. The IBD reflects the level of statistically significant intersubject connections (higher values indicate greater interaction in brain activity between subjects); in contrast, the complementary information that is provided by divisibility and modularity describes how dissociated two subnetworks are (high values of divisibility and modularity indicate that the two subnetworks, identified by the two subjects’ brains, do not interact).

We evaluated the effects of fairness level and type of agency on these indices by ANOVA. The results of the ANOVA for the four bands are reported in Table 1, which shows that only global efficiency, IBD, divisibility, and modularity were influenced by the interaction between factors (agency and fairness)—primarily in the theta, alpha (except for the modularity), and beta bands. The gamma band did not show any significant results for agency, fairness, or interaction factors, except for IBD and global efficiency of the interaction (agency*fairness). Effect size associated to the results in Table 1 is reported is supplementary materials.

**Table 1:** ANOVA on indices derived from multiple-brain networks.

		Global efficiency	Local efficiency	Path length	Clustering	IBD	Modularity	Divisibility
Theta band	agency (d.f. = 1.14)	9.78*	5.97*	31.55*	0.66	0.026	6.42*	0.35
	fairness (d.f. = 2.28)	0.51	0.22	0.24	1.76	5.69*	1.88	3.46
	agency x fairness (d.f. = 2.28)	11.39*	1.94	1.26	0.05	11.13*	4.84*	9.62*
Alpha band	agency (d.f. = 1.14)	5.64*	3.31	23.93*	0.34	0.17	10.47*	1.26
	fairness (d.f. = 2.28)	0.37	1.09	0.44	1.52	2.37	4.57*	7.54*
	agency x fairness (d.f. = 2.28)	2.09	0.57	2.05	0.92	5.52*	3.78	6.13*
Beta band	agency (d.f. = 1.14)	0.18	0.01	5.78*	0.08	0.43	2.37	1.07
	fairness (d.f. = 2.28)	0.79	0.16	0.39	1.24	0.43	0.13	0.63
	agency x fairness (d.f. = 2.28)	19.04*	1.49	2.51	2.27	20.83*	11.66*	13.16*
Gamma band	agency (d.f. = 1.14)	0.09	0.52	15.28*	0.07	0.92	0.32	0.78
	fairness (d.f. = 2.28)	0.73	0.55	0.39	1.77	0.84	1.62	2.98
	agency x fairness (d.f. = 2.28)	12.09*	1.53	2.75	1.37	9.93*	1.89	2.01

Results of repeated measures ANOVA computed on the considered graph indices in the four frequency bands. F-values are reported. Significant values (p < 0.05, FDR adjusted) are in bold and highlighted by an asterisk.

Figure 4 shows the results of the group analysis of multiple-brain connectivity patterns (panel a), in which the four graph theory indices were significant by ANOVA (panels b-e) for the theta band. Panel a shows the statistical comparison between human and PC conditions for interbrain networks in the three levels of the fairness of the dictator’s decision. The number of interbrain connections increased across the three fairness levels in the human condition, whereas the opposite pattern was observed in the PC condition. Panels b-e demonstrate how the graph indices were significantly influenced by the interaction factor fairness x agency. Specifically, in panels b and c, global efficiency and IBD rise significantly in the unfair and hyperunfair conditions versus the fair condition, when the networks are related to the human condition. In contrast, the two indices that were extracted from the networks that were related to the PC condition declined significantly along the three fairness levels. The opposite behavior was noted for the other two indices: modularity (panel d) and divisibility (panel e). Both indices decreased along the three fairness levels in the human condition but rose in the PC condition.

Similar results were obtained with regard to the multiple-brain connectivity patterns and the related graph indices in the alpha and beta bands, as shown in Figs 5 and 6.

### Using graph indices to predict punishment score

The subset of graph indices above were used as regressors for the punishment score that was assigned by the punisher during the TPP. Table 2 shows the results of the statistical regression between punishment score and global efficiency, IBD, divisibility, and modularity for the hyperunfair condition and all frequency bands. Significant regression (p < 0.05 corrected by FDR for multiple comparisons^19^) was seen primarily in the human condition in theta and alpha bands. In the gamma band, the regression was significant for modularity. In the alpha band, we observed significant regression for divisibility and modularity for the PC condition, albeit at lower values versus the human condition. All significant indices predicted the punishment score. The regression was positive for IBD and the global efficiency indices and negative for the other two indices. Effect size associated to the results in Table 2 is reported in supplementary materials. The considered indices were not able to predict punishment score in the other fairness conditions.

**Table 2:** Statistical regression between punishment score and graph theory indices during hyperunfair condition.

	Hyperunfair							
	Theta band		Alpha band		Beta band		Gamma band	
	human	PC	human	PC	human	PC	human	PC
Global efficiency	0.56*	−0.30	0.57*	−0.07	0.43	0.02	0.47	−0.17
IBD	0.70*	0.34	0.53*	0.23	0.43	0.07	0.49	−0.26
Modularity	−0.54*	−0.35	−0.78*	−0.54*	−0.17	−0.39	−0.55*	0.29
Divisibility	−0.68*	−0.25	−0.74*	−0.62*	−0.02	−0.17	−0.51	0.52

Significant correlations are highlighted in bold and denoted by an asterisk (p < 0.05 corrected by FDR).

## Discussion

To our knowledge, this report is the first multiple-brain connectivity study to investigate empathic compassion and altruistic punishment. By using a dual perspective, in which the dyadic interaction between two brains is measured simultaneously, and adapting a task that is designed specifically for prosocial behavior, we observed specific connectivity patterns in relation to various levels of fairness and agency.

Firstly, we investigated if altruistic punishment was modulated by intra- and interpersonal factors. We showed that at the behavioral level, the punisher tended to spend his own resources to punish unfair behavior against a third person, even when he was not directly affected by that unjust behavior (Fig. 3). Consistently with the altruistic punishment theory, other studies have reported similar findings on altruistic and prosocial behavior in anonymous partners^4,6,7,9,20–24^. Moreover, our data showed that prosocial behavior was accentuated when the agent who was responsible for the unfair behavior was a human dictator (compared with a nonhuman agent). This result confirms the hypothesis that third-party punishment is an efficient means of enforcing strong reciprocity and promoting cooperation^25,26^. However, empathy is a highly flexible and context-dependent response^27^. The results of our explorative correlation analysis between punishment scores and questionnaire responses (which will need to be confirmed in a larger sample), suggest that altruistic punishment is not a general human attitude but is modulated by individual differences in the degree of negative impression of the dictator and the self-altruistic propensity of the punisher. In fact, a recent study^28^ demonstrated that perceived empathy and subsequent help are not modulated exclusively by group membership but are primarily influenced by the negative impression of the suffering person. However, the low values obtained for the expected power of our data suggest taking such conclusion with caution.

We were also interested to investigate if multiple-brain connectivity is influenced by fairness and agency. Multiple-brain connectivity is a promising measure of simple and complex forms of social interaction, such as imitation, communication. and economic games^13–16,29–32^. Our dataset, to our knowledge, is the first conceived to investigate the use of modulations in multiple-brain connectivity to describe empathy between two subjects. Preliminary results on these data showed the feasibility of this approach to capture the empathic interaction between a single pair of subjects^17^. Our results here are consistent with previous hyperscanning studies on pairs of subjects interacting in social contexts that are controlled by game theory^14^ and in completely ecologic conditions, such those that are reproduced in a flight simulator^33^ where statistically significant connections were observed between subjects during the cooperation condition but nearly absent during the non-cooperative condition. The study here reported, involving a group of 32 subjects, demonstrates how multiple-brain connectivity is also sensitive to the empathic interaction between subjects. Specifically, we selected three indices to describe the properties of multiple-brain networks in terms of the interaction between two minds (the receiver and punisher): interbrain density, divisibility, and modularity, as potential predictors of cooperative behavior^15^. In our study, we always referred to a contrast between two conditions (i.e., human agent was contrasted to PC and PC was contrasted to human agent) to remove the inter-brain synchronization due to the shared environment and events^34^. In the human condition, we found a stronger integration between the receiver and punisher (expressed by high interconnection density and low divisibility and modularity) in the hyperunfair condition with respect to the fair condition. Similarly, in the PC condition, the integration was more robust for the fair versus hyperunfair condition (see Figs 4 and 5). Thus, the two conditions with the greatest integration were human hyperunfair and PC fair. We speculate that these conditions represent situations with higher emotional impact: negative emotion due to a judgment of inequity when a human agent commits a hyperunfair action^35^ and, conversely, a positive hedonic feeling due to a fortunate unexpected reward^36^ when the PC returns a fair outcome. Most studies on empathy have focused on negative emotional states, such as pain and disgust^28,37–42^, but recently, one study showed that positive events, such as vicarious reward, elicit enjoyment in individuals by observing others win in the absence of self-economic gain^43^. Our study is the first to manipulate the agency in the TPP task through human and nonhuman conditions, allowing positive and negative experiences to be evaluated. As a consequence, our results suggest greater integration between the punisher and receiver when they are likely to be more involved emotionally, irrespective of the emotional valence (injustice or vicarious reward). Obviously, only a sense of injustice can effect prosocial helping, and our participants punished the dictator exclusively after hyperunfair decisions, although some studies have reported occurrences of antisocial punishment (the tendency to punish prosocial cooperators)^44^. Sanctions enforce social norms and are based on strong negative emotions as a driver of norm enforcement^35^. Notably, we observed that the magnitude of the empathy-related multiple-brain integration predicted the extent to which our punishers subsequently engaged in altruistic helping (by penalizing the dictator at their own expense). A similar result was reported in an fMRI study by Hein and colleagues, in which the anterior insula (a key region that is involved in empathic pain) predicted the frequency of subsequent costly helping^28^.

With regard to the spectral properties that emerged from this study, our results demonstrated significant modulation of the multiple brain networks in the theta, alpha, and beta bands. Few EEG spectral studies have addressed empathy. Mu and colleagues provided evidence of the engagement of theta and alpha activity in empathy that is related to pain^45^. These bands were the most active during cooperation in various social contexts: theta activity declined during defection with respect to cooperation in the Prisoner’s Dilemma^14^, whereas frontal alpha was synchronized between musicians who were playing in an ensemble^46^.

In this work, we claim that multiple-brain connectivity, a new ecological experimental framework in social neuroscience, can be an efficient tool to study empathic experience. In fact, despite recent advances in investigating the neural mechanisms of empathy, studies on empathy have merely implemented single-person paradigms, using a “shared neural network,” based on similar activations when participants empathize with another person’s emotion and on self-experience of the emotion. The main limitation of this approach is that perceivers are examined in isolation instead of during their natural interaction with others. We have demonstrated that it is possible to use a more ecological approach toward studying complex social phenomena, such as compassion and altruistic punishment that is based on 2PN and multiple-brain connectivity. The modified TPP game that we used transcends simplified and artificial stimuli and permitted us to examine pleasing and unpleasing social contexts. Further, by eliciting altruistic punishment in a real-life interaction, the TPP allowed us to link the emphatic process to a prosocial behavior. In fact, the two processes are not independent, as supported by the connectivity indices during the compassion, predicting the consequent punishment behavior.

This study has several strengths and limitations. The precise cognitive and neural mechanisms involved during TPP are still debated. In fact, the TPP was implemented to investigate different complex social abilities, such as altruistic punishment^22,24^ but also trustworthness^47^, norm violations, legal decision making^48–50^ and it is not clear why individuals should incur the costs of punishment. Although literature consistently reported studies linking TPP to altruistic behaviour^51–54^, an alternative interpretation of our results cannot be ruled out completely. We cannot exclude that our punishers behave in order to make a good impression (to improve their reputation) or guided by an extrinsic motivation (a social norm). Considering the growing number of TPP studies, future works should identify and disentangle which distinct components are elicited during TPP by means of a theoretical model integrating all the scientific evidences provided by different studies.

Moreover, our sample only included male subjects, and thus, the results are not representative of both sexes. But evidence suggests that there are differences in the capacity to empathize between males and females^55^, necessitating studies on empathy with greater statistical power that include male and female participants or consider gender-related variables. Further, the signals were acquired by scalp EEG, the low spatial resolution of which is well known. Thus, the multiple-brain connectivity networks did not include any information on the spatial localization of the brain sources that were involved in empathy processes. Future studies should combine advanced source localization methods^56^ and multiple-brain connectivity approaches to overcome the low spatial resolution of EEG and provide a 2PN neurofunctional model of the brain circuits that constitute that basis of the empathy that is established between two subjects.

Our study is proof of concept of the use of a combination of hyperscanning and multiple-brain connectivity in examining complex social abilities. Our connectivity results are linked to behavioral performance and intra- and interpersonal psychological factors. Thus, our findings can be extended to identify the earliest signs of atypical development of the social brain in various psychological and psychiatric diseases that are affected by compromised empathy, an ability that connects people to each other.

## Methods

### Participants

A total of 21 pairs of male subjects, aged 18–30 years [mean 23.46 (SD = 3.7) and mean IQ 106.82 (SD = 13.33)], collected through the Raven SPM^57^, were enrolled. Pairs were matched with respect to age and IQ. Participants were recruited through advertisements in local schools and universities. All participants were right-handed, as determined by the Edinburgh handedness inventory^58^, with normal or corrected-to-normal vision. Psychiatric and neurological disorders were controlled by the Young adult self-report (YASR)^59^ and a medical interview. The YASR assesses emotional and behavioral problems in a standardized format with regard to internalizing (i.e., anxiety, depression) and externalizing (i.e., hyperactivity, aggression) behaviors. The broad categories of internalizing and externalizing problems and total scores were used to exclude participants with symptoms of a mental or behavioral disorder (T-scores < 60).

All participants were informed about the purpose of the experiment, and written informed consent was obtained prior to participation. All the procedures were carried out in accordance with the principles and guidelines of the Declaration of Helsinki, and all experimental protocols were approved by the ethics committee of the University of Frankfurt (Germany). A total of 15 of 21 pairs [mean age 23.69 (SD 3.2) and mean IQ 108.75 (SD 13.6)] underwent an EEG. The subjects received a lump sum payment of 20 € for participation, in addition to the money that they earned during the TPP game (range 0 to 30 €).

### Experimental design

Each pair of subjects acted out the Third Party Punishment (TPP), a novel paradigm that explores the neural mechanisms of simple forms of social interaction, such as emotional sharing, and complex social abilities, such as altruistic punishment^22,24,60^. The TPP paradigm involved three subjects: player A (the *dictator*), player B (the *receiver*), and player C (the *observer*, who in turn can act as a *punisher*). Player A had an initial endowment of 20 points, which he could share with player B. We allowed three possible conditions for such sharing to occur:

(i) Fair: 10 points to player A, 10 to player B
(ii) Unfair: 14 points to player A, 6 to player B
(iii) Hyperunfair: 18 points to player A, 2 to player B

Player A’s decision was shown to players B and C. Player C observed the dictator game and could use part, or all, of his endowment (4 points) to punish player A’s behavior. For every punishment point that was invested by player C, 3 points was subtracted from player A’s payoff, and 1 point was added to player B’s payoff: for example, if player C invested all 4 of his points (the maximum punishment) to sanction player A’s behavior, 12 points was subtracted from player A’s payoff, and 4 points was added to player B’s payoff. Details on the timeline of the paradigm are reported in Figs 1 and 2.

We focused on the empathic interaction between players B and C (the receiver and observer/punisher). For this reason, the experimental subjects were randomly assigned to these roles, whereas the dictator was played by the PC for half of the trials (PC condition) and by an actor for the other half (human condition). Further, to avoid discrepancies in the evaluation of fairness within the dyad, during the recruitment phase, each participant received a self-evaluated questionnaire in which he assessed his fairness for various combinations of a fair, unfair, and hyperunfair proposal. Consequently, each dyad (receiver and punisher) was lumped according to their evaluation of fairness.

Each dyad performed a total of 210 trials, divided into 7 blocks of 35 trials each, equally and randomly providing the three conditions (fair, unfair, and hyperunfair). All three subjects (dictator, receiver, and punisher) wore EEG caps, but data were collected only for the receiver and punisher (the dictator was a confederate).

### Questionnaires

After the EEG hyperconnectivity session, the punishers filled out the the following self-reported questionnaires: Empathy Quotient (EQ)^61^, Impression Scale (IMP-S)^62^, Altruism facet scale of the Revised NEO Personality Inventory (NEO-PI-R)^63^. A detailed description of the questionnaires and the statistical analysis used are descripted in the supplementary information.

### Statistical analysis of behavioral data

During the TPP paradigm, we tracked the punishment score, assigned from the punisher to the dictator, in the various experimental conditions. The scores were analyzed by two-way ANOVA, considering agency (human, PC) and fairness (fair, unfair, hyperunfair) as within-group factors. Newman-Keuls post hoc test was then applied to further assess the significant factors (p < 0.05).

### Data acquisition and analysis of EEG data

#### Simultaneous Multisubject EEG Recordings

The neuroelectric hyperscanning recordings were performed with a 128-channel EEG acquisition system (Brain Product GmbH, Germany - for each subject: 61 EEG + 3EOG Ag/AgCl electrodes placed according to the 10–10 EEG system, referenced to linked mastoids, ground at Fpz). The impedances were maintained below 10 kOhm. The EEG/EOG signals were collected at a sampling frequency of 250 Hz and filtered using a high-pass filter with a cutoff of 0.1 Hz. To delete sources of variance between the four amplifiers due to electrical noise and electrode impedance, the same calibration was established for devices to adjust their sensitivities and equalize the gains between devices. Because the signals of two interacting subjects were recorded as a unique system, all problems that were related to simultaneous recording of the traces were weakened.

### Preprocessing of EEG Signals

EEG signals were band-pass-filtered in the range of 1-45 Hz. Independent component analysis (ICA) was used to remove ocular artifacts. EEG traces were segmented in relation to the specific timing of the paradigm—[0 3000] ms (period of interest)—according to the onset of the window during which the dictator’s decision was shown to the other two players. Then, a semiautomatic procedure, based on a threshold ( ± 80 µV), was applied to remove residual muscular artifacts. Only artifact-free epochs that were common to both pilots were considered in the subsequent analyses. No statistical differences were found in the number of epochs between experimental conditions.

### Brain-to-brain connectivity estimation

A subset of 20 channels (Fp1, Fp2, F5, Fz, F6, FC1, FC2, T7, C3, C4, T8, CP1, CP2, P7, P3, Pz, P4, P8, O1, O2) among the 61 recorded channels was selected for each subject. The data that were recorded simultaneously for both players were jointly subjected to connectivity estimation. Specifically, we used an extension of the partial directed coherence (PDC) estimator to the multisubject case, optimized for hyperscanning purposes^12^. PDC is a spectral estimator that is based on Granger causality^64^, providing directed influences between any given pair of signals in a multivariate dataset^65,66^. The novelty of brain-to-brain connectivity lies in its construction of a multivariate autoregressive (MVAR) model as the basis of the PDC estimation of a dataset that is composed of the perfect union of EEG data that belong to two or more subjects in pair. However, because the intrinsic characteristics of EEG signals can vary widely from subject to subject, they were normalized. Data from each subject in the dyad were normalized using z-score, allowing one side to avoid spurious results due to differences in the amplitude of the two players in a dyad.

The estimated networks were then validated using asymptotic statistics^67,68^, imposing a significance level of a 5% corrected for multiple comparisons by means of false discovery rate (FDR)^19^. The accuracy of this interbrain connectivity estimator has been demonstrated in hyperscanning studies^13–15,33^.

We obtained four submatrices of model parameters: two that were related to the cortical connectivity of each individual subject and two that were related to intersubject functional links. Finally, the validated PDC values were averaged in four bands of interest—theta, 3–7 Hz; alpha, 8–12 Hz; beta, 13–29 Hz; and gamma, 30–40 Hz—and mapped on a scalp model.

### Extraction of adjacency matrices

We performed a statistical comparison (percentile test, significance level of 5% corrected by FDR^19^) between networks that were related to the human and PC conditions at the level of the single dyad to extract the corresponding adjacency matrix, on which graph indices were computed. Such In particular, two percentiles (5% and 95%, FDR corrected^19^) were extracted from the distribution over the group of connectivity networks that were related to the PC condition and used as statistical thresholds for the connectivity networks at the single-subject level. This test provides two adjacency matrices: i) one that contains the results of the right tail of the test human > PC—i.e., the connections that are significantly increased in the human versus PC condition (referred to as the human condition); ii) one that contains the results of the left tail of the test human < PC—i.e., the connections that are significantly decreased in the human versus PC condition (referred to as the PC condition). This test was repeated for each fairness level and frequency band. A total of 24 (2 types of dictator, 3 fairness levels, and 4 frequency bands) adjacency matrices were obtained for each single dyad.

### Graph theory in brain-to-brain networks

In this study, we considered the most frequently used global indices (global efficiency, local efficiency, clustering, path length) and multiple-brain-related indices (IBD, divisibility, modularity) that characterize the level of interaction between subjects^69–73^. They were computed for all networks that were obtained by contrasting PDC in the various conditions. All indices were extracted for each subject, frequency band, and experimental condition. In the Supplementary information section, a detailed description of the graph-theory indices used in this study can be found.

### Statistical analysis of EEG data

The following statistical analyses were performed:

(i) Group analysis of connectivity patterns. Statistical comparisons were performed to isolate the social aspects that were elicited by the paradigm: paired t-test at the level of the single connection (significance level of 5% corrected by FDR) between networks that is elicited during the human and PC conditions. The test was conducted separately for the three fairness levels of the dictator’s decision and the four frequency bands.
(ii) ANOVA of graph indices. The three indices (interbrain density, divisibility, and modularity) were separately analyzed by 2 × 3 repeated measures ANOVA, considering agency (2 levels: human, PC) and fairness of the dictator’s decision (3 levels: fair, unfair, hyperunfair) as the main within-group factors. Newman-Keuls post hoc test was then applied to further investigate the significant factors. The ANOVA was repeated for each frequency band. FDR correction was applied to adjust for multiple testing.
(iii) Regression between punishment score and graph indices. Linear regression was performed between graph indices and the punishment score that was assigned by the punisher to the dictator—both of which were related to the hyperunfair condition. The regression was repeated for each type of agency, frequency, and fairness level.

### Data availability

Fully anonymized data can be made available to researchers upon request regarding scientific aspects (such as data analysis).

## Electronic supplementary material

Supplementary Information
