# Supplementary material for: Multiple-Brain Connectivity During Third Party Punishment: an EEG Hyperscanning Study
Source: Sci Rep. 2018 May 1;8:6822. doi: 10.1038/s41598-018-24416-w (PMC5931604; doi:10.1038/s41598-018-24416-w)
Supplement: Supplementary file 1 — Supplementary Information [file 41598_2018_24416_MOESM1_ESM.doc]

**Multiple-Brain Connectivity During Third Party Punishment: an EEG Hyperscanning Study**

Ciaramidaro A.^1,2,$^, Toppi J.^1,3,$^, Casper C.^2^, Freitag C.M.^2^, Siniatchkin M. ^2,4^ °, Astolfi L.^1.3^ °*.

^1^Department of Computer, Control, and Management Engineering, Univ. of Rome

“Sapienza”, Rome, Italy, 00185 Italy

^2^Department of Child and Adolescent Psychiatry, Psychosomatics, and Psychotherapy,

Goethe-University, Frankfurt/M, 60528 Germany

^3^Neuroelectrical Imaging and Brain Computer Interface Laboratory, Fondazione Santa Lucia IRCCS, Rome, Italy, 00179 Italy

^4^Institute of Medical Psychology and Medical Sociology, University of Kiel, 24113 Germany

^$^ equal contribution

° equal contribution

*CORRESPONDING AUTHOR:

Prof. Laura Astolfi

Department of Computer, Control, and Management Engineering

Sapienza University of Rome

Via Ariosto 25, 00185 Rome, Italy

Phone: +39-0677274047; Fax: +39-0677274129

E-mail: laura.astolfi@uniroma1.it

**SUPPLEMENTARY INFORMATION**

**Questionnaires and statistical analysis of the questionnaires**

After the EEG hyperconnectivity session, the punishers filled out the following self-reported questionnaires:

- Empathy Quotient (EQ) is a questionnaire that measures empathy in adults (“I really enjoy caring for other people” or “I tend to get emotionally involved with a friend’s problems”)^61^. The EQ contains 40 empathy items and 20 filler/control items; on each item, a person can score 2, 1, or 0. High scores correspond to more emphatic behavior.
- Impression Scale (IMP-S)^62^. These items assess participants’ awareness and impressions of the other person (“How similar to you do you think the person is” or “How likeable did you find this person?”) on a 9-point scale. We tested for the punishers’ impressions of the dictator.
- Altruism facet scale of the Revised NEO Personality Inventory (NEO-PI-R)^63^. NEO Altruism measures active concern for the welfare of others (“I'm trying to be kind for everybody I meet” or “My first reaction is to trust people”) and is a subscale that contains 16 items that are answered on a 5-point Likert scale, ranging from strongly agree (1) to strongly disagree (5).

For one subject, the data resulted incomplete; thus, we included only the data that were related to 20 dyads in the analysis.

We were interested in correlating the punishers’ behavior with their individual differences (empathy, impression, and altruism), as revealed in the questionnaires (EQ, IMP-S, and NEO Altruism subscale). First, we tested for normal distribution using Shapiro-Wilk test. The null hypothesis of this test is that the population is normally distributed; consequently, if the p-value is greater than the chosen alpha level, then the null hypothesis that the data came from a normally distributed population cannot be rejected. For each test, the p-value was greater than 0.05; thus, we proceeded with Pearson correlation. The correlation analyses were applied to the “hyperunfair” punishment score (human and PC) and questionnaires scores (EQ, IMP-S, and NEO Altruism subscale). The correlations were performed in an exploratory manner, and no alpha correction was applied.

**Graph theory indices**

The relevant properties of multiple-brain networks can be described, quantified, and summarized by indices that are derived from the classical graph theory^69^ or defined ad hoc to address the specific properties of this type of connectivity^70^. In this study, we considered the most frequently used global indices (global efficiency, local efficiency, clustering, path length) and multiple-brain-related (IBD, divisibility, modularity) indices that characterize the level of interaction between subjects. They were computed for all networks that were obtained by contrasting PDC in the various conditions.

*Global efficiency* is the average of the inverse of the geodesic length
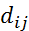
 and represents the efficiency of communication between all N nodes in a network^71^. *Local efficiency* is the average of the global efficiencies that are computed on each subgraph G_i that belongs to the network and represents the efficiency of communication between all nodes around node i in the network^71^. The *clustering coefficient* describes the intensity of interconnections between neighbors of a node and is defined as the fraction of triangles around a node or the fraction of a node’s neighbors that are neighbors of each other^72^. The *characteristic path length* is the average shortest path length in the network, wherein the shortest path length between two nodes is the minimum number of edges that must be traversed to get from one node to another^73^.

*Interbrain density* (IBD)*^70^* is an index that is specifically defined for hyperscanning applications, consisting of the number of statistically significant intersubject connectivity links for each condition, normalized to the maximum number of possible interbrain connections. The other two indices were *divisibility* and *modularity* (for details, see^15^), which provide information about the possibility of decomposing the network into two subnetworks, one for each subject in the pair. High values of divisibility and modularity are associated with situations in which the two subjects are not interacting (a low number of links that connect the two subjects), whereas low values characterize situations with strong interactions between subjects (high number of links between two subjects). In the case of a network that comprises two interacting persons, these two indices represent a measure of the level of communication between them (the lowest values for the two indices reflect the greatest interaction between them)^15^.

All indices were extracted for each subject, frequency band, and experimental condition.

**Effect size in statistical tests**

In the following tables, we report the effect sizes associated to the 2x3 repeated measures ANOVAs and to the regression between punishment score and graph theory indices performed in the study. Tab.S1 lists the values of partial eta squared parameter associated to each of the ANOVAs described in Tab.1. It is worth of note that all the ANOVAs’ significant results (with the only exception of LE in theta band for the AGENCY factor) were associated to a high effect size, categorized according to the criteria suggested by Cohen in 1992 (partial eta squared > 0.1379)^74–76^. In Tab.S2 we collected the effect size (Cohen’s f) associated to the regression analysis reported in Tab.2. Also in this case, significant regressions in Tab.2 are associated to a high effect size (> 0.4)^76^.

**Table S1. Effect size for the ANOVAs.** Partial eta squared for each of the ANOVAs reported in Tab.1. In bold, the values > 0.1379 (high effect size).

|  |  | Global  efficiency | Local efficiency | Path length | Clustering | IBD | Modularity | Divisibility |
| --- | --- | --- | --- | --- | --- | --- | --- | --- |
| Theta  band | agency (d.f. = 1.14) | **0.34** | 0.09 | **0.69** | 0.04 | 0.002 | **0.31** | 0.02 |
|  | fairness (d.f. = 2.28) | 0.03 | 0.01 | 0.01 | **0.36** | **0.29** | 0.12 | **0.2** |
|  | agency x fairness (d.f. = 2.28) | **0.44** | 0.12 | 0.08 | 0.003 | **0.44** | **0.26** | **0.41** |
| Alpha  band | agency (d.f. = 1.14) | **0.21** | **0.19** | **0.63** | 0.02 | 0.01 | **0.43** | 0.08 |
|  | fairness (d.f. = 2.28) | 0.03 | 0.07 | 0.03 | 0.1 | **0.14** | **0.25** | **0.35** |
|  | agency x fairness (d.f. = 2.28) | 0.13 | 0.04 | 0.13 | 0.06 | **0.28** | **0.21** | **0.3** |
| Beta band | agency (d.f. = 1.14) | 0.01 | 0.001 | **0.29** | 0.005 | 0.03 | **0.14** | 0.07 |
|  | fairness (d.f. = 2.28) | 0.05 | 0.01 | 0.03 | 0.08 | 0.03 | 0.01 | 0.04 |
|  | agency x fairness (d.f. = 2.28) | **0.58** | 0.09 | **0.24** | **0.23** | **0.6** | **0.45** | **0.48** |
| Gamma band | agency (d.f. = 1.14) | 0.006 | 0.03 | **0.52** | 0.005 | 0.06 | 0.02 | 0.005 |
|  | fairness (d.f. = 2.28) | 0.05 | 0.04 | 0.03 | **0.21** | 0.06 | 0.1 | **0.17** |
|  | agency x fairness (d.f. = 2.28) | **0.46** | 0.1 | **0.21** | 0.09 | **0.4** | 0.12 | 0.12 |

**Table S2. Effect size for the regression analysis.** Cohen’s *f* for each of the regressions reported in Tab.2. In bold, the values > 0.4 (high effect size).

|  | Hyperunfair | | | | | | | |
| --- | --- | --- | --- | --- | --- | --- | --- | --- |
|  | Theta band | | Alpha band | | Beta band | | Gamma band | |
|  | human | PC | human | PC | human | PC | human | PC |
| Global efficiency | **0.46** | 0.1 | **0.48** | 0.005 | 0.23 | 0.0004 | 0.28 | 0.03 |
| IBD | **0.96** | 0.13 | 0.39 | 0.06 | 0.23 | 0.005 | 0.32 | 0.07 |
| Modularity | **0.41** | 0.14 | **1.55** | **0.41** | 0.03 | 0.18 | **0.43** | 0.09 |
| Divisibility | **0.86** | 0.07 | **1.21** | **0.62** | 0.0004 | 0.03 | 0.35 | 0.37 |

References

74. Cohen, J. Statistical Power Analysis for the Behavioral Sciences. (Routledge, 1988).

75. Cohen, J. Eta-Squared and Partial Eta-Squared in Fixed Factor Anova Designs. Educ. Psychol. Meas. 33, 107–112 (1973).

76. Richardson, J. T. E. Eta squared and partial eta squared as measures of effect size in educational research. Educ. Res. Rev. 6, 135–147 (2011).
